# Bidding adieu IJP

**DOI:** 10.4103/0253-7613.59920

**Published:** 2009-12

**Authors:** Shiv Prakash

**Affiliations:** Indian Journal of Pharmacology, E-mail: drshiv@synchronresearch.com

My term as Chief Editor ends with this issue. I had a great experience as Editor of the Indian Journal of Pharmacology (IJP) for 3 years. I cherish these memorable moments. I am also feeling good about my contribution to the Society.

The new incumbent, Dr. RK Dikshit, is an able and suitable candidate to be the Chief Editor, and has served IJP as Executive Editor for 3 years. He has contributed immensely during this period and he is the right person to continue the good work. Any takeover has to be smooth and complete; we cannot and should not be starting from scratch. Starting from scratch leads to a loss of valuable data and expertise and the credits gained by the journal take a dip. A communication gap does most damage to authors, who lose out on the articles they submitted in the previous regime. Hence, I assure that the changeover is smooth and complete.

The journal needs committed reviewers. With increasing specialization and researchers getting more focused, reviewers with generic interest may not be able to do justice to certain areas of research. We have been inviting researchers, monitoring the reviewers and trying to build up the database of reviewers with varied research interests. The online system also has a facility to search and invite reviewers through PubMed from distant places. Distance, therefore, is not a deterrent if the valuable manuscripts get their due justice.

Young researchers and academicians should be motivated to publish and also review. A formal training in scientific writing and reviewing should be made mandatory in all postgraduate courses in pharmacology. Senior reviewers can train their protégés and students and initiate them into this difficult task from an early stage. The members of the current editorial team conducted three workshops on scientific writing so far, the latest one being the 28^th^ Annual Conference of Indian Pharmacological Society, Gujarat Chapter, at Karamsad.

As this journal caters to basic and clinical researchers in pharmacology, a balance needs to be maintained between the articles published. The present editorial board has tried to bridge this gap and bring in a balance by encouraging articles on drug utilisation studies, pharmacovigilance, pharmacoepidemiology and clinical research in a journal without compromising on good quality and relevant articles on basic research in pharmacology.

As the stature of the journal increases, a reverse trend of offshore researchers submitting their research work to an “Indian” journal may occur. IJP has been receiving articles from Malaysia, China, Turkey, Nigeria and Nepal. We must strive to take it farther.

The most important development for the Journal is the listing in PubMed and Science Citation Index. This has increased the global accessibility of scientific articles from IJP and credibility to the authors. This was possible only by the constant striving of our publisher, Medknow Publications, and the editorial team. I sincerely thank Dr. DK Sahu for his personal efforts in this direction. I am sure he will continue to lend the support to our Journal.

I also feel, as outgoing Chief Editor, that the quality of pharmacology research is diminishing in India. The reasons may be political or inadequate facilities in the educational institutes, or may be animal right issues. It is time that the Society steps in and does some soul-searching to discover the reasons for the decrease in good-quality pharmacology research. Pharmacology as a science has an immense role to play in the discovery of new drugs in future within India. We need liberal laws to encourage research that will eventually help in finding new drugs for many neglected diseases.

Thank you for providing me an opportunity to work with this Journal. I have learnt a lot academically and also on human and professional fronts, some the hard way and some the nice way. I thank all Executive members of the Society for giving me an opportunity to serve this great Journal. I wish the Journal a great success under the leadership of the now Chief Editor.

Good Bye

